# Somnolencia diurna excesiva en adultos mayores de un programa municipal de actividad física

**DOI:** 10.31053/1853.0605.v81.n1.41826

**Published:** 2024-03-27

**Authors:** Ivon Johana Avila Ovalle, Ana Cristina Arango Arango, Freiser Eceomo Cruz Mosquera, Nathalia Andrea Gómez-Barreiro, Yerly Alexandra Márquez-Pérez, Claudia Lorena Perlaza, Katherine Lozano Gómez, Sary Zamora-Vidal, Marcela Nayibe Garzón-Morera

**Affiliations:** 1 Universidad Santiago de Cali Colombia; 2 Universidad Santiago de Cali. Clínica Santa Barbara, Palmira, Colombia Palmira Colombia; 3 Universidad Santiago de Cali. Clínica Palmira SA, Colombia Palmira Colombia

**Keywords:** adulto mayor, actividad física, trastornos de somnolencia excesiva, sueño, older adult, physical activity, disorders of excessive somnolence, sleep, idoso, tividade física, distúrbios do sono por sonolência excessiva, sono

## Abstract

**Introducción:**

El ejercicio físico ha demostrado tener un impacto favorable en la salud del individuo. Su combinación con otros estilos de vida saludable, puede impactar de forma positiva diversas áreas entre las que se encuentra la calidad del sueño.

**Objetivo:**

Determinar la frecuencia de somnolencia diurna excesiva en adultos mayores de un programa municipal de actividad física de Santiago de Cali, Colombia.

**Metodología:**

Estudio de corte trasversal que incluyó 605 adultos mayores de un programa comunitario de la Secretaría de Recreación y Deporte de la Ciudad de Cali, Colombia, durante octubre de 2018 y junio de 2019. Para determinar la presencia de somnolencia diurna excesiva se usó la escala de somnolencia Epworth.

**Resultados:**

El 81,5% de la población era de sexo femenino con una edad promedio de 73±8 años. La prevalencia de somnolencia diurna excesiva fue del 10,5%. Sólo se encontró diferencias significativas en la frecuencia de somnolencia diurna excesiva por estrato socioeconómico, siendo más habitual en el bajo (13% vs 4% P=0,03).

**Conclusión:**

La somnolencia diurna excesiva es un fenómeno que afecta los adultos mayores, sin embargo, su frecuencia puede ser menor en aquellos que realizan actividad física.

## Abstract (en)

**Introduction:**

Physical exercise has been shown to have a favorable impact on the health of the individual. Its combination with other healthy lifestyles can positively impact various areas, including sleep quality.

**Objective:**

To determine the frequency of excessive daytime sleepiness in older adults of a municipal physical activity program in Santiago de Cali, Colombia.

**Methodology:**

Cross-sectional study that included 605 older adults from a community program of the Secretary of Recreation and Sports of the City of Cali, Colombia, during October 2018 and June 2019. To determine the presence of excessive daytime sleepiness, the Epworth Sleepiness Scale was used.

**Results:**

81.5% of the population was female with a mean age of 73±8 years. The prevalence of excessive daytime sleepiness was 10.5%. Significant differences were only found in the frequency of excessive daytime sleepiness by socioeconomic stratum, being more common in the low socioeconomic stratum (13% vs 4% P=0.03).

**Conclusion:**

Excessive daytime sleepiness is a phenomenon that affects older adults; however, its frequency may be lower in those who are physically active.

## Resumo

**Introdução:**

O exercício físico demonstrou ter um impacto favorável na saúde do indivíduo. Sua combinação com outros estilos de vida saudáveis pode impactar positivamente em diversas áreas, inclusive na qualidade do sono

**. Objetivo:**

determinar a frequência de sonolência diurna excessiva em idosos de um programa municipal de atividade física em santiago de cali, colômbia.

**Metodologia:**

estudo transversal que incluiu 605 idosos de um programa comunitário da secretaria de recreação e esportes da cidade de cali, colômbia, durante outubro de 2018 e junho de 2019. para determinar a presença de sonolência diurna excessiva, a escala de sonolência de epworth .

**Resultados:**

81,5% da população era do sexo feminino com idade média de 73±8 anos. a prevalência de sonolência diurna excessiva foi de 10,5%. diferenças significativas foram encontradas apenas na frequência de sonolência diurna excessiva por estrato socioeconômico, sendo mais comum no inferior (13% vs 4% p=0,03).

**Conclusão:**

a sonolência diurna excessiva é um fenômeno que afeta os idosos, porém sua frequência pode ser menor naqueles que praticam atividade física.

**CONCEPTOS CLAVE**

**Qué se sabe sobre el tema.**

El envejecimiento es un proceso natural que trae consigo diferentes cambios en el ser humano, uno de los más importantes está relacionado con las modificaciones del sueño, aspecto que puede impactar de manera importante las actividades de la vida diaria y la calidad de vida. Algunos autores han reportado que la prevalencia de somnolencia diurna excesiva puede alcanzar hasta el 30% de los adultos mayores.

**Qué aporta este trabajo.**

En el presente estudio, se reporta la frecuencia de somnolencia diurna excesiva en adultos mayores que asisten de manera regular a un programa de alcance municipal de la Secretaría de Recreación y Deporte de la ciudad de Santiago de Cali. Lo anterior cobra importancia, dado que la actividad física combinada con otros hábitos saludables ha mostrado tener un efecto positivo en la calidad de sueño y la salud general de los adultos mayores.

**Divulgación**

Con el envejecimiento, los adultos mayores experimentan cambios estructurales y funcionales que pueden resultar en una disminución del equilibrio en diferentes sistemas. Este deterioro progresivo, que se acentúa con el paso de los años, varía de una persona a otra y está influenciado por factores ambientales y estilos de vida. Como resultado, se producen diversas alteraciones, entre las cuales se destacan los cambios en el de sueño. A pesar de lo anterior, la adopción de hábitos de vida saludables, en lo que se incluye el ejercicio físico puede traer no sólo beneficios en la calidad de sueño, sino también en la salud en general de esta población. En el presente trabajo se estudió la frecuencia de somnolencia diurna excesiva en adultos mayores de un programa municipal de actividad física de Santiago de Cali, Colombia.

## Introducción

La Organización Mundial de la Salud (OMS) considera adulto mayor a la persona que supera los 60 años de edad ^1^ . Estos, a raíz del envejecimiento experimentan cambios estructurales y funciónale, con una consiguiente reducción del equilibrio en los diversos sistemas ^2^ . El deterioro progresivo que se hace mucho más evidente con el paso de los años, varía de un individuo a otro y está influenciado por factores ambientales y estilos de vida, generando alteraciones entre las que se encuentra la disminución en la calidad de sueño ^3^ .

Normalmente el sueño garantiza la recuperación mental y física, mejora el estado de ánimo y la memoria. La calidad del mismo está determinada factores endógenos y exógenos que desencadenan patrones de sueño corto, intermedios y largos ^4^ . Ante la presencia de una privación frecuente del sueño y despertares nocturnos, aumenta la posibilidad de interrumpir el proceso de vigilia durante el día, generando somnolencia diurna excesiva (SDE), alteración que se presenta en cerca del 27% de los adultos mayores, produciendo repercusiones en la capacidad intelectual y motora, lo que dificulta el desarrollo de las actividades de la vida diarias ^5^ .

Según Zalai et al. ^6^ la SDE está relacionada con factores como enfermedades crónicas, alteraciones del estado de ánimo, anomalías del sistema nervioso central y efectos de medicamentos, y se asocia a mayor riesgo de deterioro cognitivo y demencia senil. En el mismo sentido, Figueredo et al. ^7^ encontraron que el 65,8% del adulto mayor con SDE presentan un deterioro mental en las edades de 60 a 74 años, con evidencia de limitación en el aprendizaje y la concentración.

El ejercicio físico ha demostrado tener un impacto favorable en la salud del individuo, algunos estudios han puesto en evidencia que la actividad física combinada con la práctica de otros hábitos saludables, ayuda a disminuir la presión arterial, aumento la tolerancia a la glucosa, disminuye el riesgo de desarrollar eventos cardiovasculares, mejora el sueño y por ende disminuye la frecuencia de la somnolencia diurna excesiva ^8^ ; Lo anterior se ve reflejado en el hecho de que lo adultos mayores vinculados a programas de actividad física por lo general tienen mejor calidad de sueño y mayor desempeño en las actividades de la vida diaria que sus pares sedentarios ^8^ .

## Objetivo

Determinar la frecuencia de SDE en adultos mayores que asisten a un programa municipal de actividad física en diferentes comunas de la ciudad de Santiago de Cali, Colombia.

## Materiales y Métodos

### Diseño y población

Estudio de corte trasversal en el que participaron 605 adultos mayores vinculados a un programa comunitario de la Secretaría de Recreación y Deporte de la Ciudad de Cali durante octubre de 2018 y junio de 2019. Se incluyeron personas con edad ≥ 60 años que aceptaron voluntariamente participar del estudio; se excluyeron las personas que manifestaron incapacidad para suministrar la información y aquellas que no estaban presentes al momento de realizar la medición.

### Variables de estudio

En el estudio se contemplaron variables sociodemográficas como edad, sexo, estado civil, barrió en el que se encuentra el escenario deportivo donde asisten los participantes al programa y comunas, entendiendo el último término como las subunidades administrativas en las que se divide el área urbana de la ciudad. Adicionalmente, se incluyó el estrato socioeconómico, el cual, de acuerdo al Departamento Administrativo Nacional de Estadística de Colombia determina la clasificación de la condición económica del individuo de acuerdo con la ubicación del inmueble en el que vive, considerando mejor condición económica a mayor estrato (el menor estrato es 1 y el mayor 6). Por otro lado, se consideraron variables antropométricas como peso, talla e índice de masa corporal (IMC). Las personas se clasificaron con bajo peso cuando IMC < 18,5 kg/m^2^, peso normal (18,5 -24,9 kg/m^2^) sobrepeso (25,0-29,9 kg/m^2^) obesidad grado I (30,0 -34,5 kg/m^ 2^) obesidad grado II (35,0 -39,9 kg/m^2^) obesidad grado III (> 40 kg/m^2^) de acuerdo a lo estipulado por la OMS ^9^ .

### Instrumento de medición

Para determinar la presencia de somnolencia diurna excesiva se usó la escala de somnolencia Epworth propuesta por Johns ^10^ y validad en Colombia por Chica et al. ^11^ El instrumento consta de un total de 8 preguntas con respuestas puntuadas entre 0 y 3 (0=nunca y 3= elevada posibilidad) las cuales indagan sobre la posibilidad de quedarse dormido durante el desarrollo de 8 actividades cotidianas como leer, ver televisión, estar sentado en un lugar público, viajar en un auto, posterior al almuerzo, etc. De acuerdo con el puntaje global los sujetos fueron clasificados como normal (≤10), somnolencia excesiva mínima (11-12) somnolencia excesiva moderada (13-15) y somnolencia excesiva severa (16-24) . La información para el estudio fue recolectada por un total de 9 personas que asistieron en horas de la mañana a los escenarios deportivos donde se realizan las actividades enmarcadas en el programa.

### Plan de análisis y consideraciones éticas

El procesamiento de los datos se ejecutó en el paquete estadístico SPSS versión 24, inicialmente se realizó un análisis exploratorio de las variables para identificar valores omitidos y extremos. Para establecer la normalidad de las variables cuantitativas se utilizó la prueba de Kolmogorov Smirnov con corrección de Lilliefors. Las variables cuantitativas fueron expresadas a través de medidas de tendencia central con su respectiva medida de dispersión, adicionalmente las variables cualitativas se describieron en frecuencia y porcentajes. Para establecer la diferencia en la frecuencia de somnolencia por subgrupos se usó el test de Chi2 considerando un valor p <0,05 como estadísticamente significativo.

La investigación siguió los lineamientos éticos de la resolución 8430 de 1993 del Ministerio de salud de Colombia y de la declaración del Helsinki; el protocolo contó con el aval ético de la Universidad Santiago de Cali y la aprobación del programa Canas y Ganas de la Secretaría de Recreación y Deporte de Cali. Todos los participantes diligenciaron consentimiento informado y sus datos fueron tratados de forma confidencial.

## Resultados

El 81,5% de la población estudiada era de sexo femenino con una edad promedio de 73±8 años, la mayoría de los participantes tenían como nivel de formación "primaria" (48%) eran de estrato socioeconómico 1 a 3 (77%) y sólo un 7,5% aún se encontraban trabajando en el momento del estudio. Con relación a las variables antropométricas se evidenció un promedio de peso de 64± 11 kg, talla 1,59 ± 0,07 m e IMC 25 ±4,3 kg/m^2^. Referente a la clasificación de acuerdo con el IMC se evidencia que el 3% tenía bajo peso y el 49% estaba en la categoría sobrepeso y obesidad. Ver tabla 1.

**Tabla Nº 1:** Características sociodemográficas de los adultos mayores estudiados. (n=605)

**Variable**	**n**	**%**
Sexo		
Masculino	112	18,5
Femenino	493	81,5
Estado Civil		
Soltero	217	35,9
Casado	150	24,8
Uniòn liibre	20	3,3
Divorciado	26	4,3
Viudo	192	31,7
Nivel escolar		
Primaria	293	48
Bachillerato	186	31
Tècnico	33	5
Profesional	38	6
Especialista	4	1
Ninguno	51	9
Estrato socioeconómico		
1	23	3
2	155	26
3	290	48
4	100	17
5	24	4
6	13	2
Comuna		
1	0	0,0
2	28	4,6
3	11	1,8
4	52	8,6
5	53	8,8
6	31	5,1
7	41	6,8
8	38	6,3
9	30	5,0
10	22	3,6
11	40	6,6
12	22	3,6
13	33	5,5
14	15	2,5
15	24	4,0
16	16	2,6
17	17	2,8
18	30	5,0
19	64	10,6
20	25	4,1
21	0	0,0
22	13	2,1
23	0	0,0
24	0	0,0
Trabaja actualmente		
Si	45	7,5
No	560	92,5
	Media	DE
Edad	73	±8
IMC	25	±4,3
	n	%
Bajo peso	16	3
Peso normal	290	48
Sobrepeso	206	34
Obesidad gradoI	74	12
Obesidad gradoII	12	2
Obesidad grado III	7	1

IMC: Índice de masa corporal

Con relación a las respuestas del cuestionario Epworth, se destaca que en las 8 actividades indagadas la frecuencia de sujetos que manifestaron poca probabilidad de dormirse fue mayor. La distribución detallada de las respuestas por pregunta se evidencia en la tabla 2. A partir del puntaje de las respuestas anteriores, se estima una frecuencia de somnolencia diurna excesiva del 10,5%. La somnolencia diurna leve se presentó en el 3,8%, moderada 3,3% y severa 3,4% de la población. Ver tabla 2.

**Tabla Nº 2:** Distribución de respuestas cuestionario Epworth. ¿Con qué frecuencia se queda dormido durante las siguientes actividades?

**Item**	**Nunca n(%)**	**Escasa posibilidad n(%)**	**Moderada posibilidad n(%)**	**Alta posibilidad n(%)**
Sentado leyendo	388(64)	95(16)	76(12)	46(8)
viendo televisión	243(40)	127(21)	137(23)	98(16)
Sentado, inactivo en lugar publico	461(76)	75(12)	53(9)	16(3)
En un carrol durante una hora marcha continua	399(66)	69(11,4)	70(11,6)	67(11)
Acostado, descansando en la tarde	200(33,1)	111(18,4)	170(28,1)	123(20,4)
Sentado y conversando con alguien	531(88)	47(8)	19(3)	8(1)
Sentado, tranquilo después de un almuerzo	270(45)	106(17)	139(23)	90(15)
En un carro mientras se detiene unos minutos	524(86)	43(7)	22(4)	16(3)

Al comparar la frecuencia de somnolencia por sexo, IMC, desarrollo de actividades laborales y estrato socioeconómico, sólo se encontró diferencias significativas por estrato socioeconómico, siendo más habitual en el bajo (13% vs 4% P=0,03). Ver tabla 3.

**Tabla Nº 3:** Comparación de frecuencia SDE por subgrupos de la población a estudio

**Variable**	**n (%)**	**Valor P**
Sexo		0,3
Hombre	55(11%)	
Mujer	9(8%)	
IMC		
Peso normal	29(10%)	0,5
Sobrepeso y obesidad	35(12%)	
Estrato socioeconómico		
Bajo	59(13%)	0,003
Alto	5(4%)	
Trabaja actualmente		
Sí	7(15%)	0,2
No	57(10%)	

Peso normal: 18,5-24,9 kg/m2 Sobrepeso y obesidad: ≥ 25,0 kg/m2

Estrato bajo: 1-3 Estrato alto: 4-6

## Discusión y/o Conclusión

Los trastornos del sueño se hacen más frecuentes con el envejecimiento y a menudo limitan el desarrollo de las actividades de la vida diaria. Pese a lo anterior, estudios preliminares sugieren que la actividad física contribuye a mejorar la calidad del sueño y tiene un impacto positivo en la calidad de vida ^12, 13^ . En la presente investigación, tras estudiar adultos mayores pertenecientes a un programa municipal que incluye actividad física se encontró una frecuencia de somnolencia diurna excesiva de 10,5%.

Este hallazgo es similar, aunque ligeramente menor de lo encontrado por otros autores. Téllez et al. ^14^ en 313 adultos mayores de Monterrey y el área metropolitana de Nuevo León, evidenciaron somnolencia diurna en el 16% de la muestra. De igual forma, Vashum et al. ^15^ en una población de 3053 adultos entre 55 y 85 años en la que aplicaron la escala de somnolencia de Epworth identificaron una prevalencia de SDE del 15,3%, siendo más alta en los hombres. Es importante mencionar que, si bien la SDE en el presente estudio fue ligeramente mayor en hombres, no se hallaron diferencias estadísticamente significativas.

Por otro lado, se encontró que la SDE era más frecuente en personas de estratos socioeconómicos bajos frente a aquellas de estratos altos (13% versus 4% p= 0,003), lo que cobra sentido si se considera que el estado económico generalmente se traduce en mejores condiciones de trabajo (cuando aplica), vivienda, alimentación y por lo tanto juega un papel fundamental.

Si bien el estudio reporta la somnolencia diurna excesiva en adultos mayores expuestos a actividad física, entre las limitaciones de la investigación se encuentran la naturaleza del diseño y la ausencia de un grupo control que permitiera comparar los resultados. Para futuros estudios se sugiere considerar aspectos adicionales como la calidad de vida, la cual constituye una medida de impacto más amplia tras la exposición a la actividad física.

## References

[1] Varela Pinedo LF (2016). Salud y calidad de vida en el adulto mayor [Health and quality of life in the elderly]. Rev Peru Med Exp Salud Publica.

[2] Cruz Menor E, Hernández Rodríguez Y, Morera Rojas BP, Fernández Montequín Z, Rodríguez Benitez JC (2008). Trastornos del sueño en el adulto mayor en la comunidad. Rev. Ciencias Médicas.

[3] Moreno Reyes P, Muñoz Gutiérrez C, Pizarro Mena R, Jiménez Torres S (2020). Efectos del ejercicio físico sobre la calidad del sueño, insomnio y somnolencia diurna en personas mayores. Revisión de la literatura. Rev Esp Geriatr Gerontol.

[4] Durán Agüero S, Sánchez Reyes H, Díaz Narváez V, Araya Pérez M (2015). Factores asociados a la somnolencia diurna en adultos mayores chilenos. Rev Esp Geriatr Gerontol.

[5] Dzierzewski JM, Dautovich N, Ravyts S (2018). Sleep and Cognition in Older Adults. Sleep Med Clin.

[6] Zalai D, Bingeliene A, Shapiro C (2017). Sleepiness in the Elderly. Sleep Med Clin.

[7] Figueredo Ferrer N, Sotolongo Castillo I, Arcias Madera RC, Díaz Pita G (2003). Caracterización del adulto mayor en la comunidad. Rev Cubana Enfer.

[8] Chasens ER, Sereika SM, Weaver TE, Umlauf MG (2007). Daytime sleepiness, exercise, and physical function in older adults. J Sleep Res.

[9] Aguilar-Parra JM, Gallego J, Fernández-Campoy JM, Pérez-Gallardo ER, Trigueros R, Alías-García A, Álvarez J, Cangas AJ (2015). Influencia de programas de actividad física en la calidad del sueño de personas mayores de 55 años. Revista de Psicología del Deporte.

[10] Johns MW (1991). A new method for measuring daytime sleepiness: the Epworth sleepiness scale. Sleep.

[11] Chica-Urzola HL, Escobar-Córdoba F, Eslava-Schmalbach J (2007). Validación de la Escala de Somnolencia de Epworth. Rev Salud Publica (Bogota).

[12] Wanng F, Boros S (2021). The effect of physical activity on sleep quality: a systematic review. European Journal of Physiotherapy.

[13] Awick EA, Ehlers DK, Aguiñaga S, Daugherty AM, Kramer AF, McAuley E (2017). Effects of a randomized exercise trial on physical activity, psychological distress and quality of life in older adults. Gen Hosp Psychiatry.

[14] Téllez A, Juárez-García D, Jaime-Bernal L, García-Cadena C (2016). Prevalencia de trastornos de sueño en relación a factores sociodemográficos y depresión en adultos mayores de Monterrey, México. Revista Colombiana de Psicología.

[15] Vashum KP, McEvoy MA, Hancock SJ, Islam MR, Peel R, Attia JR, Milton AH (2015). Prevalence of and associations with excessive daytime sleepiness in an Australian older population. Asia Pac J Public Health.

